# Next-generation sequencing of cell-free microbial DNA in blood samples of critically ill children: a single-center experience

**DOI:** 10.1186/s40348-026-00226-3

**Published:** 2026-04-08

**Authors:** Jonas E. Adolph, Christina Pentek, Thomas Bauch, Clara Held, Thorsten Brenner, Ursula Felderhoff-Müser, Silke Grumaz, Petra Horvatek, Mathis Steindor, Lucia Asar, Sebastian Voigt, Jan Dziobaka, Christian Dohna-Schwake, Sarah C. Goretzki

**Affiliations:** 1 Department of Pediatrics I, Neonatology, Pediatric Intensive Care, Pediatric Neurology, Pediatric Infectious Diseases, Essen University Hospital, Hufelandstr. 55, Essen, D-45122 Germany; 2 Department of Anesthesiology and Intensive Care Medicine, Essen University Hospital, Essen, Germany; 3 Noscendo GmbH, Duisburg, Germany; 4 Department of Pediatric Pulmonology and Sleep Medicine, Essen University Hospital, Essen, Germany; 5 University Duisburg-Essen, Institute for Virology, Essen, Germany; 6 University Duisburg-Essen, Institute of Medical Microbiology, Essen, Germany

**Keywords:** Cell-free DNA, Metagenomic next-generation sequencing, Pediatric sepsis, Infectious disease diagnostics, Antimicrobial stewardship

## Abstract

**Background:**

Rapid and accurate pathogen detection is critical for optimizing outcomes in pediatric sepsis. Next-generation sequencing (NGS) of cell-free DNA (cfDNA) from blood enables culture-independent identification of microbial DNA from bacteria, viruses, fungi, and parasites. We evaluated the diagnostic yield and clinical impact of cfDNA-based NGS in critically ill and predominantly immunocompromised pediatric patients (≤ 18 years) with suspected infection.

This retrospective single-center study included pediatric patients who underwent plasma cfDNA-NGS at a tertiary care hospital in Germany. Following computational removal of human DNA, remaining sequences were aligned to curated microbial reference databases. Diagnostic performance was compared with blood cultures and viral PCR, and clinical relevance was assessed by pediatric infectious disease specialists.

**Results:**

111 tests in 78 pediatric patients, mostly with systemic inflammatory response syndrome of unknown etiology, were performed. Overall, 61 tests (54.5%) were positive for pathogenic cfDNA. Compared with conventional microbiological diagnostics, NGS demonstrated a sensitivity of 64.7% and specificity of 88.2% when blood cultures and viral PCR served as the reference standard. NGS identified additional pathogens in a substantial proportion (41.1%) of cases that remained negative by standard testing. Of those pathogens only found by NGS, over 60% were deemed clinically relevant. In 14.8% of positive NGS results, a pathogen-specific therapy was started, while 40.2% of tests led to a discontinuation of therapy (51.0% of negative tests). Out of all positive NGS, 38 (62.3%) were classified as clinically relevant. NGS testing also detected rare infections with fungi and parasites in four cases each.

**Conclusion:**

Detection of pathogenic cfDNA through NGS from blood shows promising results as an additional diagnostic tool in critically ill pediatric patients with suspected infections. Clinical utility is currently still limited by its high cost, undetermined diagnostic validity and limitations in testing for resistances and restricted availability of raw sequencing data due to data-protection constraints.

**Supplementary Information:**

The online version contains supplementary material available at 10.1186/s40348-026-00226-3.

## Background

Diagnostic evaluation is particularly challenging in pediatrics due to age-dependent physiological responses, limited ability of young children to localize symptoms, and frequent overlap of infectious and non-infectious causes of systemic inflammation, and subsequently for patient outcome. Additionally, invasive sampling is often restricted by blood volume limitations and clinical instability, especially in infants and critically ill children [1, 2]. Children and adolescents with complex underlying conditions, impaired immune responses, or ongoing inflammation frequently receive multiple anti-infective treatments prior to or during hospitalization. These prior exposures may obscure the clinical picture and lead to false-negative microbiological results.

Conventional diagnostic approaches include culture-based techniques, nucleic acid amplification methods such as polymerase chain reaction (PCR), and antigen detection assays. Each of these methods has distinct limitations. In children, limited available blood volume can reduce the sensitivity of blood cultures, while repeated sampling may not be feasible. Furthermore, long turnaround times can delay antimicrobial optimization, increasing exposure to broad-spectrum therapy.

Culture-based diagnostics are time-consuming and susceptible to pre-analytical limitations such as contamination, delayed processing, or insufficient sample volumes, which may be particularly relevant in pediatrics due to restricted blood volumes and frequent use of small-volume blood culture bottles [3, 4]. PCR and antigen-based tests, while faster, are typically limited to predefined pathogens and provide little or no information on antimicrobial resistance [5–7]. Consequently, pathogens may remain unidentified or the time to diagnosis may delay effective clinical decision-making.

Recent advances in molecular diagnostics have introduced next generation sequencing (NGS) of microbial genetic material circulating freely in the bloodstream as a promising complementary approach. This technology enables culture-independent identification of bacterial, viral, fungal, and parasitic DNA fragments in a single assay. In adult cohorts, plasma cfDNA sequencing has been reported to increase pathogen detection compared with standard-of-care microbiology and may provide actionable results within 24–48 h, supporting earlier targeted antimicrobial therapy in selected cases [8–14]. In pediatrics, early studies and cohort reports suggest potential clinical utility, particularly in immunocompromised children, but data remain heterogeneous and limited regarding diagnostic accuracy, interpretation criteria, and real-world impact on antimicrobial management [15–18].

The aim of this study was to evaluate the diagnostic yield, clinical relevance, and potential therapeutic impact of NGS for detection of circulating microbial DNA in pediatric patients with suspected infection at a tertiary care center. We hypothesised that NGS would identify additional pathogens not detected by conventional methods and thereby influence clinical management decisions.

## Materials and methods

### Study design and participants

This retrospective, single-center study evaluated all results from Next Generation Sequencing (NGS) of circulating cell-free deoxyribonucleic acid (cfDNA) obtained from pediatric patients (≤ 18 years) treated at the University Hospital Essen, Germany, between January 2020 and January 2023. All pediatric patients (≤ 18 years) who underwent plasma cfDNA-NGS during the study period were included. No additional exclusion criteria were applied. Since NGS testing was requested at the discretion of the treating team in consultation with pediatric infectious diseases specialists, this cohort represents a clinically selected population with suspected infection and therefore reflects real-world use rather than systematic screening.

Indications for NGS testing included systemic inflammatory response syndrome (SIRS) of unknown origin, suspected infection by rare or atypical pathogens, and follow-up analysis during ongoing treatment. Indications were not defined in a prospective protocol but followed local clinical practice and were determined case-by-case after interdisciplinary discussion.

Testing was performed at the discretion of the attending physician and after consultation with the pediatric infectious disease (ID) team. All NGS results were subsequently reviewed for clinical relevance by the same team. In total, 78 patients and 111 NGS tests were included.

Clinical data collected comprised demographic information, primary diagnosis, clinical presentation, results of standard microbiological diagnostics, and inflammatory parameters such as C-reactive protein (CRP) and procalcitonin (PCT) when available.

### Sample collection and DNA extraction

Up to 10 mL of peripheral whole blood was drawn under aseptic conditions into two Streck Cell-Free DNA BCT CE tubes (Streck, Omaha, NE, USA) containing a cell- and cfDNA stabiliser. Samples were stored at 4 °C and transported within 24 h to the laboratory facility of Noscendo GmbH (Reutlingen, Germany) for processing. Blood volume was adapted to patient age and clinical condition. In younger children and infants, smaller volumes were occasionally used, which may have reduced analytical sensitivity in individual cases. Plasma was separated by centrifugation at 1,600 × g for 10 min at 4 °C, followed by centrifugation of the supernatant at 16,000 × g for 10 min at 4 °C. cfDNA was extracted using the QIAsymphony DSP Circulating DNA Kit on the QIAsymphony SP instrument (Qiagen, Hilden, Germany). DNA concentration and purity were assessed with the Qubit dsDNA HS Assay Kit (Thermo Fisher Scientific, Waltham, MA, USA).

### Library preparation and sequencing

Sequencing libraries were prepared from 1 ng of cfDNA. Single-read sequencing (1 × 75 bp) was performed using Illumina NextSeq 1000/2000 instruments (Illumina, San Diego, CA, USA) with Illumina reagents. Each run included up to 14 multiplexed samples and at least 24 million reads per sample were required. Single-end sequencing was used as part of the standardized CE-IVD workflow provided by the diagnostic platform. While paired-end sequencing may improve alignment confidence in some settings, single-end reads at high depth and intermediate length allow sensitive detection of microbial cfDNA while supporting rapid turnaround and standardized clinical reporting. The minimum threshold of 24 million reads per sample was specified by the diagnostic provider after extensive validations as a quality control criterion to ensure sufficient sequencing depth for clinical reporting. The minimum threshold of 24 million reads per sample was specified by the diagnostic provider as a quality control criterion to ensure sufficient sequencing depth for clinical reporting.

### Bioinformatic analysis

Bioinformatic processing was conducted using the CE-IVD-certified DISQVER^®^ platform (Version R4 and R5, Noscendo GmbH, Duisburg, Germany). Human reads were computationally excluded. Remaining non-human sequences were systematically compared against a proprietary microbial genome database containing over 16,000 microbial species and 1,500 pathogenic taxa (defined as curated “species-level entries and pathogenic taxa” according to vendor documentation).

### Statistical analysis

Statistical analyses were performed using R software (version 4.3.1; R Core Team, 2023). Continuous variables were summarised as medians with interquartile ranges (IQR). The Wilcoxon rank-sum test was used for comparisons of continuous variables between groups. The chi-squared test assessed independence between categorical variables. Correlations between continuous variables were analysed using Pearson’s correlation coefficient, and 95% confidence intervals (CI) were calculated. A two-sided p-value < 0.05 was considered statistically significant.

### Ethical approval

This study was approved by the Ethics Committee of the University of Duisburg-Essen (approval number 23-11277-BO). The requirement for informed consent was waived due to the retrospective design and anonymized data analysis. All procedures were performed in accordance with the Declaration of Helsinki and relevant institutional guidelines.

## Results

### Patient characteristics

Between November 2020 and January 2023, a total of 111 tests were performed on 78 patients. Per patient, one to nine tests were performed. The median age was 10.3 years (range: 0.5–17.6). Most patients (79.3%, *n* = 89) had an underlying chronic disease, and 67 (60.4%) were immunocompromised. Patient demographics as well as the results of standard diagnostics are presented in Table 1.

**Table 1:** Demographic and clinical characteristics

Characteristic	Overall *N* = 111^1^	negative NGS *N* = 51^1^	positive NGS *N* = 60^1^
Median age; years	10.3 (4.5, 12.9)	8.6 (3.4, 12.5)	11.2 (5.3, 13.7)
C-reactive protein, mg/dl	5.0 (1.4, 11.1)	3.0 (0.7, 10.6)	6.0 (2.0, 15.1)
Procalcitonin, ng/ml	0.2 (0.0, 1.7)	0.2 (0.0, 1.0)	0.1 (0.0, 2.6)
Antibiotic therapy at time of NGS	66 (60%)	26 (52%)	40 (67%)
Underlying diagnosis			
Immune compromised	24 (22%)	9 (18%)	15 (25%)
Oncological	43 (39%)	15 (29%)	28 (47%)
Other^1^	21 (19%)	12 (24%)	9 (15%)
None	23 (21%)	15 (29%)	8 (13%)
Indication for NGS-testing			
SIRS of unknown origin	96 (86%)	44 (86%)	52 (87%)
Suspected infection with a rare germ	12 (11%)	4 (7.8%)	8 (13%)
Control	3 (2.7%)	3 (5.9%)	0 (0%)
Blood-cultures			
Positive	7 (8.4%)	2 (5.1%)	5 (11%)
Negative	76 (92%)	37 (95%)	39 (89%)
Urine-cultures			
Positive	10 (18%)	4 (14%)	6 (21%)
Negative	47 (82%)	25 (86%)	22 (79%)
PCR detection of virus in blood			
Positive	15 (52%)	4 (27%)	11 (79%)
Negative	14 (48%)	11 (73%)	3 (21%)
Broncho-alveolar lavage			
Positive	2 (67%)	0 (0%)	2 (100%)
Negative	1 (33%)	1 (100%)	0 (0%)
Clinical impact			
Confirmation of diagnosis	7 (6.3%)	2 (3.9%)	5 (8.3%)
Therapy control	29 (26%)	6 (12%)	23 (38%)
Discontinuation of anti-infective therapy	45 (41%)	26 (51%)	19 (32%)
No start of anti-infective therapy	33 (30%)	19 (37%)	14 (23%)
Start of specific anti-infective therapy	8 (7.2%)	0 (0%)	8 (13%)

Diagnoses classified under others were: JIA, cystic fibrosis, BPD, Morbus Crohn, unknown congenital disorder, epileptic encephalopathy, myopathy, hypoxic-ischaemic encephalopathy, short bowel syndrome, sickle cell anemia

^1^Median (Q1, Q3); n (%)

### Indications and testing

NGS was requested mostly for systemic inflammatory response syndrome of unknown origin (96/111; 86.5%). Additional indications included suspected infection with rare pathogens (12/111; 10.8%) and follow-up analysis (29/111; 26.1%). Eight patients underwent follow-up testing; overlap between indications was possible. At the time of sampling, 67 patients (60.4%) were receiving at least one antibiotic therapy. We assessed antibiotic exposure as a binary variable (ongoing therapy vs. none) at sampling. Detailed durations were not analyzed systematically, which may have influenced cfDNA detectability.

### NGS results

In total, 60 tests (54.1%) were positive for microbial cfDNA. The median number of pathogens detected per test was one (interquartile range [IQR] 1–2, range 1–5). Among positive samples, at least one bacterium was found in 41 (68.3%), one virus in 24 (40%), one fungus in 4 (6.7%), and one parasite in 4 (6.7%) samples. Mixed bacterial–viral findings occurred in seven tests (11.5%). NGS identified additional pathogens in 41.4% (46/111) of tests where conventional diagnostics in blood were negative. The complete list of microorganisms detected by NGS is presented in Table 2*and Supplements Table 4*.

**Table 2:** Microbes identified by NGS; Numbers indicate counts of detections across tests. Group A = bacteria, Group B = viruses, Group C = fungi, Group D = parasites

Group	Species
Bacteria	*Enterococcus faecium* (14), *Staphylococcus epidermidis* (5), *Burkholderia contaminans* (3), *Haemophilus influenzae* (3), *Escherichia coli* (2), *Fusobacterium nucleatum* (2), *Pseudomonas aeruginosa* (2), *Serratia marcescens* (2), *Bacteroides fragilis* (1), *Bacteroides stercoris* (1), *Cutibacterium acnes* (1), *Flavonifractor plautii* (1), *Haemophilus parainfluenzae* (1), *Klebsiella pneumoniae* (1), *Kocuria palustris* (1), *Lactobacillus sakei* (1), *Moraxella osloensis* (1), *Mycobacterium chimaera* (1), *Parabacteroides merdae* (1), *Parvimonas micra* (1), *Phocaeicola vulgatus* (1), *Prevotella oris* (1), Propionibacterium sp. oral taxon 193 (1), *Pseudomonas protegens* (1), *Staphylococcus aureus* (1), *Staphylococcus hominis* (1), *Streptococcus gordonii* (1)
Viruses	Human gammaherpesvirus 4 (8), Human betaherpesvirus 5 (7), Human mastadenovirus c (5), Torque teno virus 10 (5), Human polyomavirus 1 (4), Human alphaherpesvirus 3 (2), Human betaherpesvirus 6b (2), Torque teno virus 13 (2), Torque teno virus 19 (2), Adenovirus 1 (1), Adenovirus 5 (1), Torque teno virus 11 (1), Torque teno virus 16 (1), Torque teno virus 22 (1), Torque teno virus 24 (1)
Fungi	*Aspergillus fumigatus* (3), *Candida dubliniensis* (1)
Parasites	*Enterocytozoon bieneusi* (3), *Leishmania donovani* (1), *Leishmania infantum* (1)

### Comparison with standard diagnostics

When compared with conventional blood diagnostics for bacteria and viruses, NGS showed a sensitivity of 64.7%, specificity of 88.2%, positive predictive value (PPV) of 18.0% and a negative predictive value (NPV) of 88.2%. When only clinically relevant pathogens were considered, sensitivity increased to 87.5%, specificity was 66.7%, PPV 18.4%, and NPV 66.7%. Among seven cases with positive blood cultures, NGS detected the same pathogen in four (57.1%), while three were missed. In urine cultures, one of four positive bacterial findings (25%) was confirmed by NGS. For viral pathogens identified by PCR, NGS fully matched four of fifteen (26.7%) and partially matched three of fifteen (20%). All four fungal pathogens were confirmed by conventional diagnostics. Zero cases were found in which NGS was negative but classical diagnostics were positive. Detailed contingency tables comparing NGS and conventional results are provided in Table 3.

**Table 3:** Comparison of NGS with conventional diagnostics. Panels A = bacteria; B = viruses; C = only relevant bacteria; D = only relevant viruses

	A - Only Bacteria		B - Only Viruses	
	Conventional diagnostics positive	Conventional diagnostics negative	Conventional diagnostics positive	Conventional diagnostics negative
NGS positive	4	38	7	17
NGS negative or different pathogen	3	67	5	83

	C - Only Relevant Bacteria*		D - Only Relevant Viruses*	
NGS positive	3	22	4	11
NGS negative or different pathogen	1	14	1	24

*Relevant organisms were defined as NGS-detected species judged clinically significant based on infection focus, patient context, and inflammatory response by pediatric infectious disease specialist, excluding typical contaminants or commensals

The low PPV is mainly explained by the fact that conventional blood-based diagnostics (blood culture and viral PCR) were used as the reference standard and may miss pathogens detected by cfDNA-NGS, particularly under ongoing antimicrobial therapy or when pathogens are localized outside the bloodstream. Consequently, several additional NGS-only detections were classified as false positives in the statistical comparison, although a proportion of these were deemed clinically relevant.

### Factors influencing NGS positivity

The likelihood of a positive NGS result was not significantly associated with serum C-reactive protein (*p* = 0.13) or procalcitonin (*p* = 0.97) levels. Antibiotic treatment at the time of testing also did not influence the probability of NGS positivity (*p* = 0.15). Correlation analysis showed no relationship between inflammatory markers and sequencing read counts (CRP: *r* = − 0.09, 95% CI − 0.34 to 0.17, *p* = 0.49; PCT: *r* = − 0.05, 95% CI − 0.37 to 0.28, *p* = 0.78).

### Clinical impact of NGS results

NGS findings led to initiation of pathogen-specific therapy in 12 cases (10.8%), confirmation of existing diagnoses in 8 cases (7.1%), and discontinuation of antibiotic therapy in 45 cases (40.5%). Among the 60 positive NGS results, 37 (62.7%) were judged clinically relevant by the infectious disease team. Clinically relevant organisms included *Aspergillus fumigatus*,* Burkholderia contaminans*,* Candida dubliniensis*,* Enterocytozoon bieneusi*,* Fusobacterium nucleatum*,* Leishmania infantum*,* Mycobacterium chimaera*, and Pseudomonas protegens. Clinical relevance was defined by consensus assessment of two pediatric infectious diseases specialists considering clinical presentation, plausibility of the organism as a pathogen, concordance with other diagnostics, host risk factors (e.g., immunosuppression), and whether the result led to targeted diagnostic confirmation or treatment adaptation. Pathogens classified as clinically irrelevant were mainly Torque teno viruses and Propionibacterium species. Those clinically irrelevant findings were documented within the patient chart, discussed with the clinical team, but generally did not prompt treatment changes.

## Discussion

We present a cohort of 78 pediatric patients treated at a tertiary care center with a total of 111 NGS analyses for pathogenic cfDNA, mostly in cases of SIRS of unknown origin.

Compared with conventional microbiological diagnostics, NGS demonstrated a sensitivity of 64.7% and specificity of 88.2% when blood cultures and viral PCR served as the reference standard. Taking conventional diagnostics as the gold-standard, the positive predictive value was modest, as NGS identified additional pathogens in a substantial proportion of cases that were negative by standard testing and therefore were calculated as false-positives, lowering the positive predictive value. Of those pathogens only found by NGS, over 60% were deemed clinically relevant. In clinical practice, interpretation requires differentiation between true pathogens, colonizers, transient DNAemia, and contaminants. Without careful clinical correlation, detection of clinically irrelevant organisms may lead to unnecessary antimicrobial escalation. Our approach of structured infectious diseases review aimed to mitigate this risk.

Our results align with previous studies in adult and pediatric populations, which also demonstrated that NGS can broaden the diagnostic yield and expedite pathogen identification [14–16, 19–22]. Similar pediatric cohorts report that cfDNA-NGS can identify uncommon or unexpected pathogens, particularly in immunocompromised hosts [15–18]. Our study expands these findings by providing a European single-center experience and describing several rare fungal and parasitic detections that triggered targeted confirmatory diagnostics and treatment adaptations. This particularly applies to children with chronic diseases or those receiving immunosuppressive therapy, who constituted the main subgroup within our cohort, as also reported elsewhere [17, 18]. The turnaround time from drawing of blood to receipt of NGS results was one to two days, with the longest timespan being four days due to weekends or holidays, which is similar to previously reported times in a clinical setting [14, 15, 22]. A turnaround time of 1–2 days is clinically relevant for escalation, targeted treatment, or de-escalation decisions in critically ill children once initial empiric therapy has been started. However, we did not quantify time-to-antibiotic-modification or compare decision-making timelines formally. Thus, NGS results were mostly available faster than blood culture results and were similar in timing to viral PCR assays. Given that the outcome of pediatric sepsis depends critically on prompt and accurate therapy, rapid pathogen detection and identification may be lifesaving [1–3].

Importantly, negative NGS results, if shown to have adequate diagnostic reliability, may support safe de-escalation or discontinuation of antimicrobial treatment, thereby reducing unnecessary exposure, toxicity, and the risk of secondary infections such as *Clostridioides difficile* [23].

Positive NGS results identified several rare pathogens which were subsequently only detected by conventional diagnostics after targeted investigations based on the NGS findings were initiated. Without NGS overlooked pathogens included *Burkholderia contaminans*, *Enterocytozoon bieneusi*, *Fusobacterium nucleatum*, *Leishmania infantum* and *donovani*, *Mycobacterium chimaera*. Rare pathogens like *Enterocytozoon bieneusi*, can be challenging to detect using standard microbiological techniques [24]. Similarly, *Mycobacterium chimaera* often requires prolonged culture and specialized laboratory protocols for identification [25]. Meanwhile, *Leishmania infantum*, which is endemic to specific regions, may be overlooked in non-endemic settings unless clinical suspicion prompts targeted testing [26]. In the presented patient cohort, the ability of NGS to detect such pathogens not only enhanced diagnostic accuracy but also had direct therapeutic implications, enabling the timely initiation of targeted antimicrobial or antiparasitic therapies.

Possible explanations for non-detection in culture-positive cases include low pathogen cfDNA fraction, insufficient sequencing depth in those runs, high host-to-microbe DNA ratio, different sample materials used in conventional diagnostics (such as urine) and timing of sampling after antimicrobial exposure. These interventions significantly impacted patient outcomes and might have otherwise been delayed or entirely missed.

Besides its short turnaround time and broad range of detectable pathogens, further advantages of NGS diagnostics include detecting resistance factors within the same sample and workflow [27, 28], as well as its use in different specimens, such as cerebrospinal fluid, lavages or tissues [29, 30].

This study has several limitations. First, it was conducted at a single tertiary center and primarily included chronically ill and immunocompromised patients, introducing selection bias and limiting generalisability. Second, NGS testing was performed at the discretion of treating clinicians, which may have favoured patients with higher pre-test probability of infection [31]. Third, the retrospective design and relatively small sample size restricted the ability to draw definitive conclusions about diagnostic accuracy and clinical outcomes and due to the retrospective design and heterogeneous underlying diseases, we did not perform a formal outcome analysis (e.g., length of stay) attributable to NGS-guided decisions, but can comment that only two patients died. Future prospective studies should incorporate standardized outcome endpoints to evaluate clinical benefit beyond diagnostic yield.

Finally, NGS, like other sequencing-based methods, remains susceptible to contamination, and data protection regulations prevented the public release of raw sequencing data. Only aggregated results could be made available upon reasonable request.

## Conclusions

In this study, we evaluated the diagnostic yield, clinical relevance, and therapeutic impact of Next Generation Sequencing (NGS) for detection of circulating microbial DNA in pediatric patients with suspected infection at a tertiary care center. Overall, NGS results led to a documented change in antimicrobial management in 57/111 tests (51.4%), including initiation of targeted therapy (12/111, 10.8%) and discontinuation of therapy (45/111, 40.5%), whereas 8/111 tests (7.1%) mainly confirmed an existing working diagnosis without altering therapy. These findings indicate that NGS can provide meaningful diagnostic and therapeutic value when used as a complementary tool to standard microbiological testing, particularly in complex or critically ill children. However, the clinical utility of NGS remains limited by factors such as high cost, restricted availability, lack of standardized interpretation criteria, and uncertain diagnostic validity in broader populations. Future multicenter, prospective studies should focus on defining patient groups that benefit most from NGS, validating its performance metrics, and integrating resistance gene detection and quantitative analyses into clinical workflows.

## Supplementary Information

Supplementary Material 1

## Data Availability

The datasets generated and analysed during the current study are not publicly available due to patient confidentiality and data protection regulations. De-identified aggregated data may be made available from the corresponding author upon reasonable request and pending approval by the local Ethics Committee and data protection office.
