# Supplementary material for: Next-generation sequencing of cell-free microbial DNA in blood samples of critically ill children: a single-center experience
Source: Mol Cell Pediatr. 2026 Apr 8;13:12. doi: 10.1186/s40348-026-00226-3 (PMC13062146; doi:10.1186/s40348-026-00226-3)
Supplement: Supplementary file 1 — Supplementary Material 1 [file 40348_2026_226_MOESM1_ESM.docx]

**Supplements**

**Table 4. Clinically relevant pathogens detected by cfDNA-NGS without confirmation by standard blood diagnostics (“NGS-only detections”)**

| Age (years) | Immunocompromised (Y/N) | Clinical syndrome | NGS result | Conventional testing (blood) | Targeted confirmed NGS (Y/N) | Therapy changes category (stop/start/confirm/No change) | Final clinical interpretation |
| --- | --- | --- | --- | --- | --- | --- | --- |
| 13.9 | N | SIRS of unknown origin | Negative | Negative | Y | Stop | Likely clinically relevant |
| 3.0 | Y | SIRS of unknown origin | Negative | Negative | N | Confirm/No change | Likely irrelevant / uncertain |
| 9.7 | Y | SIRS of unknown origin | Negative | Negative | N | Stop | Likely clinically relevant |
| 1.6 | Y | SIRS of unknown origin | Negative | Negative | N | Stop | Likely clinically relevant |
| 2.0 | N | SIRS of unknown origin | *Fusobacterium nucleatum* | Negative | Y | Start | Likely clinically relevant |
| 2.0 | N | SIRS of unknown origin | *Fusobacterium nucleatum* | Negative | N | Adjust/continue | Likely clinically relevant |
| 2.0 | N | SIRS of unknown origin | *Fusobacterium nucleatum* | Negative | N | Stop | Likely clinically relevant |
| 15.5 | N | SIRS of unknown origin | Human Gammaherpesvirus 4 | Negative | Y | Confirm/No change | Likely irrelevant / uncertain |
| 10.3 | Y | SIRS of unknown origin | Adenovirus | Adenovirus | Y | Start | Likely clinically relevant |
| 10.3 | Y | SIRS of unknown origin | Adenovirus | Adenovirus | N | Start | Likely clinically relevant |
| 10.4 | Y | SIRS of unknown origin | Adenovirus | Adenovirus | Y | Adjust/continue | Likely clinically relevant |
| 10.8 | Y | SIRS of unknown origin | Adenovirus | Adenovirus | Y | Confirm/No change | Likely irrelevant / uncertain |
| 7.7 | N | SIRS of unknown origin | Negative | Negative | Y | Confirm/No change | Likely irrelevant / uncertain |
| 11.5 | N | SIRS of unknown origin | Negative | Negative | Y | Stop | Likely clinically relevant |
| 11.4 | Y | SIRS of unknown origin | Negative | Human Gammaherpesvirus 4 | N | Stop | Likely clinically relevant |
| 14.2 | Y | SIRS of unknown origin | Negative | Negative | Y | Stop | Likely clinically relevant |
| 4.0 | Y | SIRS of unknown origin | Human Betaherpesvirus 6 | *Klebsiella pneumoniae* with blood culture | N | Confirm/No change | Likely irrelevant / uncertain |
| 7.9 | N | Suspected infection with a rare germ | Negative | Negative | N | Confirm/No change | Likely irrelevant / uncertain |
| 3.8 | Y | SIRS of unknown origin | Human Betaherpesvirus 6 | Negative | Y | Confirm/No change | Likely irrelevant / uncertain |
| 3.9 | Y | SIRS of unknown origin | Human Betaherpesvirus 6 | Negative | Y | Stop | Likely clinically relevant |
| 4.5 | N | SIRS of unknown origin | Negative | Negative | N | Stop | Likely clinically relevant |
| 9.2 | N | SIRS of unknown origin | *Burkholderia contaminans* | Negative | N | Confirm/No change | Likely irrelevant / uncertain |
| 9.3 | N | SIRS of unknown origin | *Burkholderia contaminans* | Negative | N | Confirm/No change | Likely irrelevant / uncertain |
| 9.5 | N | SIRS of unknown origin | *Burkholderia contaminans* | Negative | Y | Confirm/No change | Likely irrelevant / uncertain |
| 11.1 | N | SIRS of unknown origin | Negative | Negative | Y | Stop | Likely clinically relevant |
| 2.0 | N | SIRS of unknown origin | Negative | Negative | N | Stop | Likely clinically relevant |
| 12.5 | Y | SIRS of unknown origin | Negative | Negative | Y | Stop | Likely clinically relevant |
| 11.1 | N | SIRS of unknown origin | Negative | Negative | Y | Stop | Likely clinically relevant |
| 3.1 | Y | SIRS of unknown origin | Negative | Human Gammaherpesvirus 5 and 6 | Y | Confirm/No change | Likely irrelevant / uncertain |
| 11.2 | Y | SIRS of unknown origin | *Enterococcus faecium, Candida krusei* | Negative | Y | Confirm/No change | Likely irrelevant / uncertain |
| 11.3 | Y | SIRS of unknown origin | *Enterococcus faecium* | Negative | Y | Confirm/No change | Likely irrelevant / uncertain |
| 11.3 | Y | SIRS of unknown origin | *Enterococcus faecium* | Negative | Y | Stop | Likely clinically relevant |
| 11.3 | Y | SIRS of unknown origin | *Enterococcus faecium* | Negative | N | Confirm/No change | Likely irrelevant / uncertain |
| 11.4 | Y | Suspected infection with a rare germ | *Enterococcus faecium* | Negative | Y | Confirm/No change | Likely irrelevant / uncertain |
| 11.4 | Y | Suspected infection with a rare germ | *Enterococcus faecium* | Negative | Y | Confirm/No change | Likely irrelevant / uncertain |
| 11.5 | Y | Suspected infection with a rare germ | *Enterococcus faecium* | Negative | Y | Stop | Likely clinically relevant |
| 11.5 | Y | Suspected infection with a rare germ | *Enterococcus faecium* | Negative | N | Confirm/No change | Likely irrelevant / uncertain |
| 11.6 | Y | Suspected infection with a rare germ | *Enterococcus faecium* | Neagtive | N | Confirm/No change | Likely irrelevant / uncertain |
| 11.6 | Y | Suspected infection with a rare germ | *Enterococcus faecium* | Negative | Y | Confirm/No change | Likely irrelevant / uncertain |
| 11.6 | Y | Suspected infection with a rare germ | Negative | Negative | Y | Confirm/No change | Likely irrelevant / uncertain |
| 11.7 | Y | Suspected infection with a rare germ | Negative | Negative | Y | Confirm/No change | Likely irrelevant / uncertain |
| 11.9 | Y | Suspected infection with a rare germ | Negative | Negative | Y | Confirm/No change | Likely irrelevant / uncertain |
| 8.5 | Y | SIRS of unknown origin | Adenovirus, Human Gammaherpesvirus 4 | Human Gammaherpesvirus 4 | N | Start | Likely clinically relevant |
| 3.0 | N | SIRS of unknown origin | Negative | Negative | Y | Confirm/No change | Likely irrelevant / uncertain |
| 5.3 | N | SIRS of unknown origin | Negative | Negative | Y | Stop | Likely clinically relevant |
| 0.5 | N | SIRS of unknown origin | Negative | Negative | Y | Stop | Likely clinically relevant |
| 12.5 | N | SIRS of unknown origin | Negative | Negative | N | Confirm/No change | Likely irrelevant / uncertain |
| 12.7 | N | SIRS of unknown origin | Negative | Negative | Y | Confirm/No change | Likely irrelevant / uncertain |
| 5.4 | Y | SIRS of unknown origin | Negative | Negative | Y | Stop | Likely clinically relevant |
| 5.5 | N | SIRS of unknownorigin | Negative | Negative | Y | Stop | Likely clinically relevant |
| 12.6 | N | SIRS of unknown origin | Negative | Negative | Y | Confirm/No change | Likely irrelevant / uncertain |
| 5.2 | Y | SIRS of unknown origin | Negative | Negative | N | Confirm/No change | Likely irrelevant / uncertain |
| 5.4 | Y | SIRS of unknown origin | Negative | Negative | N | Confirm/No change | Likely irrelevant / uncertain |
| 5.4 | N | SIRS of unknown origin | Negative | Negative | N | Stop | Likely clinically relevant |
| 10.9 | Y | SIRS of unknown origin | Negative | Negative | N | Stop | Likely clinically relevant |
| 17.3 | N | SIRS of unknown origin | *Escherichia coli* | *Escherichia coli* | Y | Confirm/No change | Likely irrelevant / uncertain |
| 2.9 | Y | SIRS of unknown origin | Torque teno virus, Human Polyomavirus 1 | Negative | Y | Confirm/No change | Likely irrelevant / uncertain |
| 3.1 | Y | SIRS of unknown origin | Torque teno virus, Human Polyomavirus 1 | *Respiratory Syncytial Virus, Boccavirus* | N | Confirm/No change | Likely irrelevant / uncertain |
| 15.9 | Y | SIRS of unknown origin | Human Alphaherpesvirus 3 | Negative | N | Confirm/No change | Likely irrelevant / uncertain |
| 13.8 | Y | SIRS of unknown origin | *Candida dubliniensis* | Negative | Y | Confirm/No change | Likely irrelevant / uncertain |
| 4.4 | Y | SIRS of unknown origin | Negative | Negative | N | Confirm/No change | Likely irrelevant / uncertain |
| 4.5 | Y | SIRS of unknown origin | Negative | Streptococcus. epidermidis, Adenovirus | Y | Stop | Likely clinically relevant |
| 2.1 | N | SIRS of unknown origin | Negative | Negative | Y | Stop | Likely clinically relevant |
| 5.1 | Y | SIRS of unknown origin | Negative | Negative | N | Confirm/No change | Likely irrelevant / uncertain |
| 17.6 | Y | SIRS of unknown origin | Negative | Negative | N | Stop | Likely clinically relevant |
| 0.9 | N | SIRS of unknown origin | Negative | Negative | Y | Confirm/No change | Likely irrelevant / uncertain |
| 3.0 | N | SIRS of unknown origin | Negative | Negative | Y | Confirm/No change | Likely irrelevant / uncertain |
| 3.4 | N | SIRS of unknown origin | Negative | Negative | N | Stop | Likely clinically relevant |
| 8.5 | N | Suspected infection with a rare germ | *Mycobacterium chimaera* | *Mycobacterium chimaera* | Y | Start | Likely clinically relevant |
| 8.6 | N | Control | Negative | *Mycobacterium chimaera* | Y | Start | Likely clinically relevant |
| 8.8 | N | Control | Negative | Negative | N | Stop | Likely clinically relevant |
| 5.4 | N | SIRS of unknown origin | Negative | Negative | N | Stop | Likely clinically relevant |
| 14.8 | N | SIRS of unknown origin | Negative | Negative | N | Stop | Likely clinically relevant |
| 13.0 | Y | SIRS of unknown origin | Negative | Negative | N | Start | Likely clinically relevant |
| 4.3 | Y | SIRS of unknown origin | Negative | Negative | N | Stop | Likely clinically relevant |
| 0.6 | Y | SIRS of unknown origin | Negative | Negative | Y | Confirm/No change | Likely irrelevant / uncertain |
| 0.7 | Y | SIRS of unknown origin | Negative | Negative | Y | Confirm/No change | Likely irrelevant / uncertain |
| 8.6 | Y | SIRS of unknown origin | *Pseudomonas protegens, Pseudomonas aeruginosa* | Negative | Y | Start | Likely clinically relevant |
| 6.1 | N | SIRS of unknown origin | Negative | Negative | Y | Confirm/No change | Likely irrelevant / uncertain |
| 14.3 | Y | SIRS of unknown origin | Negative | Negative | N | Stop | Likely clinically relevant |
| 1.6 | Y | SIRS of unknown origin | Adenovirus | *Staphylococcus. hominis* | N | Start | Likely clinically relevant |
| 16.6 | N | SIRS of unknown origin | Negative | Negative | Y | Stop | Likely clinically relevant |
| 13.9 | N | SIRS of unknown origin | Negative | Negative | Y | Stop | Likely clinically relevant |
| 17.0 | Y | SIRS of unknown origin | Negative | Human Gammaherpesvirus 4 | Y | Stop | Likely clinically relevant |
| 9.2 | N | SIRS of unknown origin | Negative | Negative | Y | Stop | Likely clinically relevant |
| 7.7 | N | SIRS of unknown origin | Negative | Negative | Y | Confirm/No change | Likely irrelevant / uncertain |
| 12.9 | N | SIRS of unknown origin | Negative | Human Gammaherpesvirus 1 | Y | Confirm/No change | Likely irrelevant / uncertain |
| 14.3 | Y | SIRS of unknown origin | Negative | Negative | Y | Stop | Likely clinically relevant |
| 2.3 | Y | SIRS of unknown origin | Negative | Negative | Y | Confirm/No change | Likely irrelevant / uncertain |
| 13.0 | N | SIRS of unknown origin | Negative | Negative | Y | Stop | Likely clinically relevant |
| 14.5 | Y | SIRS of unknown origin | Negative | Negative | Y | Stop | Likely clinically relevant |
| 15.6 | N | SIRS of unknown origin | Negative | Negative | N | Stop | Likely clinically relevant |
| 13.9 | N | SIRS of unknown origin | *Leishmania infantum, Leishmania donovani* | Negative | Y | Start | Likely clinically relevant |
| 5.3 | Y | SIRS of unknown origin | Negative | Negative | Y | Confirm/No change | Likely irrelevant / uncertain |
| 15.8 | N | Control | Negative | Negative | Y | Stop | Likely clinically relevant |
| 10.7 | Y | SIRS of unknown origin | Negative | Negative | Y | Stop | Likely clinically relevant |
| 1.6 | Y | SIRS of unknown origin | Negative | Negative | Y | Stop | Likely clinically relevant |
| 2.3 | Y | SIRS of unknown origin | Negative | *Eterococcus faecalis* | Y | Confirm/No change | Likely irrelevant / uncertain |
| 6.1 | Y | SIRS of unknown origin | Negative | Human Gammaherpesvirus 4 | Y | Confirm/No change | Likely irrelevant / uncertain |
| 13.7 | Y | SIRS of unknown origin | *Enterocytozoon bieneusi* | Negative | Y | Start | Likely clinically relevant |
| 13.8 | Y | SIRS of unknown origin | *Enterocytozoon bieneusi* | Negative | Y | Adjust/continue | Likely clinically relevant |
| 13.7 | Y | SIRS of unknown origin | *Enterocytozoon bieneusi* | Human Gammaherpesvirus 1 and 4 | Y | Adjust/continue | Likely clinically relevant |
| 13.7 | Y | SIRS of unknown origin | *Aspergillus fumigatus* | Negative | Y | Stop | Likely clinically relevant |
| 13.7 | Y | SIRS of unknown origin | *Aspergillus fumigatus* | Negative | Y | Stop | Likely clinically relevant |
| 15.3 | Y | SIRS of unknown origin | Human Gammaherpesvirus 4 | Negative | Y | Start | Likely clinically relevant |
| 14.7 | N | Suspected infection with a rare germ | Negative | Negative | Y | Stop | Likely clinically relevant |
| 14.6 | Y | SIRS of unknown origin | Negative | Negative | Y | Stop | Likely clinically relevant |
| 5.9 | Y | SIRS of unknown origin | Negative | Negative | Y | Confirm/No change | Likely irrelevant / uncertain |
| 6.6 | Y | SIRS of unknown origin | Negative | Negative | Y | Stop | Likely clinically relevant |
| 12.1 | Y | SIRS of unknown origin | Negative | Negative | Y | Confirm/No change | Likely irrelevant / uncertain |
| 12.1 | Y | SIRS of unknown origin | Negative | Negative | Y | Confirm/No change | Likely irrelevant / uncertain |

Standard diagnostics were defined as culture and viral PCR from blood. Targeted confirmatory tests included organism-specific PCR, antigen testing, microscopy, or culture from non-blood compartments as clinically indicated.

*AB = antibiotics; Y = yes; N = no;
